# Composite Likelihood Methods Based on Minimum Density Power Divergence Estimator

**DOI:** 10.3390/e20010018

**Published:** 2017-12-31

**Authors:** Elena Castilla, Nirian Martín, Leandro Pardo, Konstantinos Zografos

**Affiliations:** 1 Department of Statistics and O.R. I, Complutense University of Madrid, 28040 Madrid, Spain; 2 Department of Statistics and O.R. II, Complutense University of Madrid, 28003 Madrid, Spain; 3 Department of Mathematics, University of Ioannina, 45110 Ioannina, Greece

**Keywords:** composite likelihood, maximum composite likelihood estimator, Wald test statistic, composite minimum density power divergence estimator, Wald-type test statistics

## Abstract

In this paper, a robust version of the Wald test statistic for composite likelihood is considered by using the composite minimum density power divergence estimator instead of the composite maximum likelihood estimator. This new family of test statistics will be called Wald-type test statistics. The problem of testing a simple and a composite null hypothesis is considered, and the robustness is studied on the basis of a simulation study. The composite minimum density power divergence estimator is also introduced, and its asymptotic properties are studied.

## 1. Introduction

It is well known that the likelihood function is one of the most important tools in classical inference, and the resultant estimator, the maximum likelihood estimator (MLE), has nice efficiency properties, although it has not so good robustness properties.

Tests based on MLE (likelihood ratio test, Wald test, Rao’s test, etc.) have, usually, good efficiency properties, but in the presence of outliers, the behavior is not so good. To solve these situations, many robust estimators have been introduced in the statistical literature, some of them based on distance measures or divergence measures. In particular, density power divergence measures introduced in [1] have given good robust estimators: minimum density power divergences estimators (MDPDE) and, based on them, some robust test statistics have been considered for testing simple and composite null hypotheses. Some of these tests are based on divergence measures (see [2,3]), and some others are used to extend the classical Wald test; see [4,5,6] and the references therein.

The classical likelihood function requires exact specification of the probability density function, but in most applications, the true distribution is unknown. In some cases, where the data distribution is available in an analytic form, the likelihood function is still mathematically intractable due to the complexity of the probability density function. There are many alternatives to the classical likelihood function; in this paper, we focus on the composite likelihood. Composite likelihood is an inference function derived by multiplying a collection of component likelihoods; the particular collection used is a conditional determined by the context. Therefore, the composite likelihood reduces the computational complexity so that it is possible to deal with large datasets and very complex models even when the use of standard likelihood methods is not feasible. Asymptotic normality of the composite maximum likelihood estimator (CMLE) still holds with the Godambe information matrix to replace the expected information in the expression of the asymptotic variance-covariance matrix. This allows the construction of composite likelihood ratio test statistics, Wald-type test statistics, as well as score-type statistics. A review of composite likelihood methods is given in [7]. We have to mention at this point that CMLE, as well as the respective test statistics are seriously affected by the presence of outliers in the set of available data.

The main purpose of the paper is to introduce a new robust family of estimators, namely, composite minimum density power divergence estimators (CMDPDE), as well as a new family of Wald-type test statistics based on the CMDPDE in order to get broad classes of robust estimators and test statistics.

In Section 2, we introduce the CMDPDE, and we provide the associated estimating system of equations. The asymptotic distribution of the CMDPDE is obtained in Section 2.1. Section 2.2 is devoted to the definition of a family of Wald-type test statistics, based on CMDPDE, for testing simple and composite null hypotheses. The asymptotic distribution of these Wald-type test statistics is obtained, as well as some asymptotic approximations to the power function. A numerical example, presented previously in [8], is studied in Section 3. A simulation study based on this example is also presented (Section 3), in order to study the robustness of the CMDPDE, as well as the performance of the Wald-type test statistics based on CMDPDE. Proofs of the results are presented in the Appendix A.

## 2. Composite Minimum Density Power Divergence Estimator

We adopt here the notation by [9], regarding the composite likelihood function and the respective CMLE. In this regard, let $\left\{f\left(\cdot ;\theta \right),\theta \in \Theta \subseteq R^{p},p\geq 1\right\}$ be a parametric identifiable family of distributions for an observation y, a realization of a random *m*-vector Y. In this setting, the composite density based on *K* different marginal or conditional distributions has the form:

$$\mathrm{CL}\left(\theta ,y\right)=\prod_{k=1}^{K}{\left(f_{A_{k}}\left(y_{j},j\in A_{k};\theta \right)\right)}^{w_{k}}$$

and the corresponding composite log-density has the form:

$$c\ell \left(\theta ,y\right)=\sum_{k=1}^{K}w_{k}\ell_{A_{k}}\left(\theta ,y\right),$$

with:

$$\ell_{A_{k}}\left(\theta ,y\right)=\log f_{A_{k}}\left(y_{j},j\in A_{k};\theta \right),$$

where ${\left\{A_{k}\right\}}_{k=1}^{K}$ is a family of random variables associated either with marginal or conditional distributions involving some $y_{j}$ and j∈{1,…,m} and $w_{k}$, k=1,…,K are non-negative and known weights. If the weights are all equal, then they can be ignored. In this case, all the statistical procedures produce equivalent results.

Let $y_{1},\ldots ,y_{n}$ also be independent and identically distributed replications of y. We denote by:

$$c\ell \left(\theta ,y_{1},\ldots ,y_{n}\right)=\sum_{i=1}^{n}c\ell \left(\theta ,y_{i}\right)$$

the composite log-likelihood function for the whole sample. In complete accordance with the classical MLE, the CMLE, ${\hat{\theta }}_{c}$, is defined by:

(1) $${\hat{\theta }}_{c}=\underset{\theta \in \Theta }{\arg\max}\sum_{i=1}^{n}c\ell \left(\theta ,y_{i}\right)=\underset{\theta \in \Theta }{\arg\max}\sum_{i=1}^{n}\sum_{k=1}^{K}w_{k}\ell_{A_{k}}\left(\theta ,y_{i}\right).$$

It can also be obtained by solving the equations.

(2) $$u\left(\theta ,y_{1},\ldots ,y_{n}\right)=0_{p},$$

where:

$$u\left(\theta ,y_{1},\ldots ,y_{n}\right)=\frac{\partial c\ell (\theta ,y_{1},\ldots ,y_{n})}{\partial \theta }=\sum_{i=1}^{n}\sum_{k=1}^{K}w_{k}\frac{\partial \ell_{A_{k}}\left(\theta ,y_{i}\right)}{\partial \theta }.$$

We are going to see how it is possible to get the CMLE, ${\hat{\theta }}_{c},$ on the basis of the Kullback–Leibler divergence measure. We shall denote by $g\left(y\right)$ the density generating the data with the respective distribution function denoted by *G*. The Kullback–Leibler divergence between the density function $g\left(y\right)$ and the composite density function CL(θ,y) is given by:

$$\begin{array}{ccc} d_{KL}\left(g\left(.\right),\mathrm{CL}\left(\theta ,.\right)\right) & = & \int_{R^{m}}g\left(y\right)\log\frac{g(y)}{\mathrm{CL}(\theta ,y)}dy\\ & = & \int_{R^{m}}g\left(y\right)\log g\left(y\right)dy-\int_{R^{m}}g\left(y\right)\log\mathrm{CL}\left(\theta ,y\right)dy. \end{array}$$

The term:

$$\int_{R^{m}}g\left(y\right)\log g\left(y\right)dy$$

can be removed because it does not depend on θ; hence, we can define the following estimator of θ, based on the Kullback–Leibler divergence:

$${\hat{\theta }}_{KL}=\arg\min_{\theta }d_{KL}\left(g\left(.\right),\;\mathrm{CL}\left(\theta ,.\right)\right)$$

or equivalently:

(3) $$\begin{array}{ccc} {\hat{\theta }}_{KL} & = & \arg\min_{\theta }\left(-\int_{R^{m}}g\left(y\right)\log\mathrm{CL}\left(\theta ,y\right)dy\right)\\ & = & \arg\min_{\theta }\left(-\int_{R^{m}}\log\mathrm{CL}\left(\theta ,y\right)dG\left(y\right)\right). \end{array}$$

If we replace in (3) the distribution function *G* by the empirical distribution function $G_{n}$, we have:

$$\begin{array}{ccc} {\hat{\theta }}_{KL} & = & \arg\min_{\theta }\left(-\int_{R^{m}}\log\mathrm{CL}\left(\theta ,y\right)dG_{n}\left(y\right)\right)\\ & = & \arg\min_{\theta }\left(-\frac{1}{n}\sum_{i=1}^{n}c\ell \left(\theta ,y_{i}\right)\right) \end{array}$$

and this expression is equivalent to Expression (1). Therefore, the estimator ${\hat{\theta }}_{KL}$ coincides with the CMLE. Based on the previous idea, we are going to introduce, in a natural way, the composite minimum density power divergence estimator (CMDPDE).

The CMLE, ${\hat{\theta }}_{c}$, obeys asymptotic normality (see [9]) and in particular:

$$\sqrt{n}\left({\hat{\theta }}_{c}-\theta \right)\underset{n\to \infty }{\overset{L}{⟶}}N\left(0,{\left(G_{*}\left(\theta \right)\right)}^{-1}\right),$$

where $G_{*}\left(\theta \right)$ denotes the Godambe information matrix, defined by:

$$G_{*}\left(\theta \right)=H\left(\theta \right){\left(J(\theta )\right)}^{-1}H\left(\theta \right),$$

with H(θ) being the sensitivity or Hessian matrix and J(θ) being the variability matrix, defined, respectively, by:

$$\begin{array}{cc} H(\theta ) & =E_{\theta }\left[-\frac{\partial }{\partial \theta }u{\left(\theta ,Y\right)}^{T}\right],\\ J(\theta ) & =Var_{\theta }\left[u\left(\theta ,Y\right)\right]=E_{\theta }\left[u\left(\theta ,Y\right)u{\left(\theta ,Y\right)}^{T}\right], \end{array}$$

where the superscript *T* denotes the transpose of a vector or a matrix.

The matrix J(θ) is nonnegative definite by definition. In the following, we shall assume that the matrix H(θ) is of full rank. Since the component score functions can be correlated, we have H(θ)≠J(θ). If cℓ(θ,y) is a true log-likelihood function, then $H\left(\theta \right)=J\left(\theta \right)=I_{F}\left(\theta \right)$, $I_{F}\left(\theta \right)$ being the Fisher information matrix of the model. Using multivariate version of the Cauchy–Schwarz inequality, we have that the matrix $G_{*}\left(\theta \right)-I_{F}\left(\theta \right)$ is non-negative definite, i.e., the full likelihood function is more efficient than any other composite likelihood function (cf. [10], Lemma 4A).

We are now going to proceed to the definition of the CMDPDE, which is based on the density power divergence measure, defined as follows. For two densities *p* and *q* associated with two *m*-dimensional random variables, respectively, the density power divergence (DPD) between *p* and *q* was defined in [1] by:

(4) $$d_{\beta }\left(p,q\right)=\int_{R^{m}}\left\{q{\left(y\right)}^{1+\beta }-\left(1+\frac{1}{\beta }\right)q{\left(y\right)}^{\beta }p\left(y\right)+\frac{1}{\beta }\;p{\left(y\right)}^{1+\beta }\right\}dy,$$

for β>0, while for β=0, it is defined by:

$$\lim_{\beta \to 0}d_{\beta }\left(p,q\right)=d_{KL}\left(p,q\right).$$

For β=1, Expression (4) reduces to the $L_{2}$ distance:

$$L_{2}\left(p,q\right)=\int_{R^{m}}{\left(q(y)-p(y)\right)}^{2}dy.$$

It is also interesting to note that (4) is a special case of the so-called Bregman divergence $\int \left[T\left(p\left(y\right)\right)-T\left(q\left(y\right)\right)-\{p\left(y\right)-q\left(y\right\}T^{\prime }\left(q\left(y\right)\right)\right]dy$. If we consider $T\left(l\right)=l^{1+\beta }$, we get β times $d_{\beta }\left(p,q\right)$. The parameter β controls the trade-off between robustness and asymptotic efficiency of the parameter estimates (see the Simulation Section), which are the minimizers of this family of divergences. For more details about this family of divergence measures, we refer to [11].

In this paper, we are going to consider DPD measures between the density function $g\left(y\right)$ and the composite density function CL(θ,y), i.e.,

(5) $$d_{\beta }\left(g\left(.\right),\mathrm{CL}\left(\theta ,.\right)\right)=\int_{R^{m}}\left\{\mathrm{CL}{\left(\theta ,y\right)}^{1+\beta }-\left(1+\frac{1}{\beta }\right)\mathrm{CL}{\left(\theta ,y\right)}^{\beta }g\left(y\right)+\frac{1}{\beta }\;g{\left(y\right)}^{1+\beta }\right\}dy$$

for β>0, while for β=0, we have,

$$\lim_{\beta \to 0}d_{\beta }\left(g\left(.\right),\mathrm{CL}\left(\theta ,.\right)\right)=d_{KL}\left(g\left(.\right),\mathrm{CL}\left(\theta ,.\right)\right).$$

The CMDPDE, ${\hat{\theta }}_{c}^{\beta },$ is defined by:

$${\hat{\theta }}_{c}^{\beta }=\arg\min_{\theta \;\in \Theta }d_{\beta }\left(g\left(.\right),\mathrm{CL}\left(\theta ,.\right)\right).$$

The term:

$$\int_{R^{m}}\;g{\left(y\right)}^{1+\beta }dy$$

does not depend on θ, and consequently, the minimization of (5) with respect to θ is equivalent to minimizing:

$$\int_{R^{m}}\left(\mathrm{CL}{\left(\theta ,y\right)}^{1+\beta }-\left(1+\frac{1}{\beta }\right)\mathrm{CL}{\left(\theta ,y\right)}^{\beta }g\left(y\right)\right)dy$$

or:

$$\int_{R^{m}}\mathrm{CL}{\left(\theta ,y\right)}^{1+\beta }dy-\left(1+\frac{1}{\beta }\right)\int_{R^{m}}\mathrm{CL}{\left(\theta ,y\right)}^{\beta }dG\left(y\right).$$

Now, we replace the distribution function *G* by the empirical distribution function $G_{n}$, and we get:

(6) $$\int_{R^{m}}\mathrm{CL}{\left(\theta ,y\right)}^{1+\beta }dy-\left(1+\frac{1}{\beta }\right)\frac{1}{n}\sum_{i=1}^{n}\mathrm{CL}{\left(\theta ,y_{i}\right)}^{\beta }.$$

As a consequence, for a fixed value of β, the CMDPDE of θ can be obtained by minimizing the expression given in (6); or equivalently, by maximizing the expression:

(7) $$\frac{1}{n\beta }\sum_{i=1}^{n}\mathrm{CL}{\left(\theta ,y_{i}\right)}^{\beta }-\frac{1}{1+\beta }\int_{R^{m}}\mathrm{CL}{\left(\theta ,y\right)}^{1+\beta }dy.$$

Under the differentiability of the model, the maximization of the function in Equation (7) leads to an estimating system of equations of the form:

(8) $$\frac{1}{n}\sum_{i=1}^{n}\mathrm{CL}{\left(\theta ,y_{i}\right)}^{\beta }\frac{\partial c\ell (\theta ,y_{i})}{\partial \theta }-\int_{R^{m}}\frac{\partial c\ell (\theta ,y)}{\partial \theta }\mathrm{CL}{\left(\theta ,y\right)}^{1+\beta }dy=0.$$

The system of Equations (8) can be written as:

(9) $$\frac{1}{n}\sum_{i=1}^{n}\mathrm{CL}{\left(\theta ,y_{i}\right)}^{\beta }u\left(\theta ,y_{i}\right)-\int_{R^{m}}u\left(\theta ,y\right)\mathrm{CL}{\left(\theta ,y\right)}^{1+\beta }dy=0.$$

and the CMDPDE ${\hat{\theta }}_{c}^{\beta }$ of θ is obtained by the solution of (9). For β=0 in (9), we have:

$$\frac{1}{n}\sum_{i=1}^{n}u\left(\theta ,y\right)-\int_{R^{m}}u\left(\theta ,y\right)\mathrm{CL}\left(\theta ,y\right)dy.$$

but:

$$\int_{R^{m}}u\left(\theta ,y\right)\mathrm{CL}\left(\theta ,y\right)dy=\frac{\partial }{\partial \theta }\mathrm{CL}\left(\theta ,y\right)dy=0$$

and we recover the estimating equation for the CMLE, ${\hat{\theta }}_{c}$, presented in (2).

### 2.1. Asymptotic Distribution of the Composite Minimum Density Power Divergence Estimator

Equation (9) can be written as follows:

$$\frac{1}{n}\sum_{i=1}^{n}\Psi_{\beta }\left(y_{i},\theta \right)=0$$

with:

$$\Psi_{\beta }\left(y_{i},\theta \right)=\mathrm{CL}{\left(\theta ,y_{i}\right)}^{\beta }u\left(\theta ,y_{i}\right)-\int_{R^{m}}u\left(\theta ,y\right)\mathrm{CL}{\left(\theta ,y\right)}^{1+\beta }dy.$$

Therefore, the CMDPDE, ${\hat{\theta }}_{c}^{\beta },$ is an M-estimator. In this case, it is well known (cf. [12]) that the asymptotic distribution of ${\hat{\theta }}_{c}^{\beta }$ is given by:

$$\sqrt{n}\left({\hat{\theta }}_{c}^{\beta }-\theta \right)\underset{n\to \infty }{\overset{L}{⟶}}N\left(0,{\left(H_{\beta }\left(\theta \right)\right)}^{-1}J_{\beta }\left(\theta \right){\left(H_{\beta }\left(\theta \right)\right)}^{-1}\right),$$

being:

$$H_{\beta }\left(\theta \right)=E_{\theta }\left[-\frac{\partial \Psi_{\beta }\left(Y,\theta \right)}{\partial \theta^{T}}\right]$$

and:

$$J_{\beta }\left(\theta \right)=E_{\theta }\left[\Psi_{\beta }\left(Y,\theta \right)\Psi_{\beta }{\left(Y,\theta \right)}^{T}\right].$$

We are going to establish the expressions of $H_{\beta }\left(\theta \right)$ and $J_{\beta }\left(\theta \right).$ In relation to $H_{\beta }\left(\theta \right)$, we have:

$$\begin{array}{ccc} \frac{\partial \Psi_{\beta }\left(y,\theta \right)}{\partial \theta^{T}} & = & \beta \mathrm{CL}{\left(\theta ,y\right)}^{\beta -1}\mathrm{CL}\left(\theta ,y\right)u{\left(\theta ,y\right)}^{T}u\left(\theta ,y\right)+\mathrm{CL}{\left(\theta ,y\right)}^{\beta }\frac{\partial u{\left(\theta ,y\right)}^{T}}{\partial \theta }\\ & & -\int_{R^{m}}\frac{\partial u{\left(\theta ,y\right)}^{T}}{\partial \theta }\mathrm{CL}{\left(\theta ,y\right)}^{1+\beta }dy-\left(1+\beta \right)\int_{R^{m}}\mathrm{CL}{\left(\theta ,y\right)}^{\beta }\mathrm{CL}\left(\theta ,y\right)u{\left(\theta ,y\right)}^{T}u\left(\theta ,y\right)dy \end{array}$$

and:

(10) $$H_{\beta }\left(\theta \right)=E_{\theta }\left[-\frac{\partial \Psi_{\beta }\left(Y,\theta \right)}{\partial \theta^{T}}\right]=\int_{R^{m}}\mathrm{CL}{\left(\theta ,y\right)}^{\beta +1}u{\left(\theta ,y\right)}^{T}u\left(\theta ,y\right)dy.$$

In relation to $J_{\beta }\left(\theta \right)$, we have,

$$\begin{array}{ccc} \Psi_{\beta }\left(Y,\theta \right)\Psi_{\beta }{\left(Y,\theta \right)}^{T} & = & \left(\mathrm{CL}{\left(\theta ,y\right)}^{\beta }u\left(\theta ,y\right)-\int_{R^{m}}u\left(\theta ,y\right)\mathrm{CL}{\left(\theta ,y\right)}^{1+\beta }dy\right)\\ & & \left(\mathrm{CL}{\left(\theta ,y\right)}^{\beta }u{\left(\theta ,y\right)}^{T}-\int_{R^{m}}u{\left(\theta ,y\right)}^{T}\mathrm{CL}{\left(\theta ,y\right)}^{1+\beta }dy\right)\\ & = & \mathrm{CL}{\left(\theta ,y\right)}^{2\beta }u\left(\theta ,y\right)u{\left(\theta ,y\right)}^{T}-\mathrm{CL}{\left(\theta ,y\right)}^{\beta }u\left(\theta ,y\right)\int_{R^{m}}u{\left(\theta ,y\right)}^{T}\mathrm{CL}{\left(\theta ,y\right)}^{1+\beta }dy\\ & & -\mathrm{CL}{\left(\theta ,y\right)}^{\beta }u{\left(\theta ,y\right)}^{T}\int_{R^{m}}u\left(\theta ,y\right)\mathrm{CL}{\left(\theta ,y\right)}^{1+\beta }dy\\ & & +\left(\int_{R^{m}}u\left(\theta ,y\right)\mathrm{CL}{\left(\theta ,y\right)}^{1+\beta }dy\right)\left(\int_{R^{m}}u{\left(\theta ,y\right)}^{T}\mathrm{CL}{\left(\theta ,y\right)}^{1+\beta }dy\right). \end{array}$$

Then,

(11) $$\begin{array}{ccc} J_{\beta }\left(\theta \right) & = & E_{\theta }\left[\Psi_{\beta }\left(Y,\theta \right)\Psi_{\beta }{\left(Y,\theta \right)}^{T}\right]=\int_{R^{m}}\mathrm{CL}{\left(\theta ,y\right)}^{2\beta +1}u\left(\theta ,y\right)u{\left(\theta ,y\right)}^{T}dy \end{array}$$

(12) $$\begin{array}{c} -\int_{R^{m}}\mathrm{CL}{\left(\theta ,y\right)}^{\beta +1}u\left(\theta ,y\right)dy\int_{R^{m}}u{\left(\theta ,y\right)}^{T}\mathrm{CL}{\left(\theta ,y\right)}^{1+\beta }dy. \end{array}$$

Based on the previous results, we have the following theorem.

**Theorem** **1.** *Under suitable regularity conditions, we have:*

$$\sqrt{n}\left({\hat{\theta }}_{c}^{\beta }-\theta \right)\underset{n\to \infty }{\overset{L}{⟶}}N\left(0,{\left(H_{\beta }\left(\theta \right)\right)}^{-1}J_{\beta }\left(\theta \right){\left(H_{\beta }\left(\theta \right)\right)}^{-1}\right),$$

*where the matrices $H_{\beta }\left(\theta \right)$ and $J_{\beta }\left(\theta \right)$ were defined in (10) and (11), respectively.*

**Remark** **1.** *If we apply the previous theorem for β=0, then we get the CMLE, and the asymptotic variance covariance matrix coincides with the Godambe information matrix because:*

$$H_{\beta }\left(\theta \right)=H\left(\theta \right)\;\mathrm{and}\;J_{\beta }\left(\theta \right)=J\left(\theta \right),$$

*for β=0.*

### 2.2. Wald-Type Tests Statistics Based on the Composite Minimum Power Divergence Estimator

Wald-type test statistics based on MDPDE have been considered with excellent results in relation to the robustness in different statistical problems; see for instance [4,5,6].

Motivated by those works, we focus in this section on the definition and the study of Wald-type test statistics, which are defined by means of CMDPDE estimators instead of MDPDE estimators. In this context, if we are interested in testing:

(13) $$H_{0}:\theta =\theta_{0}\;\mathrm{against}\;H_{1}:\theta \neq \theta_{0},$$

we can consider the family of Wald-type test statistics:

(14) $$W_{n,\beta }^{0}=n{\left({\hat{\theta }}_{c}^{\beta }-\theta_{0}\right)}^{T}{\left({\left(H_{\beta }\left(\theta_{0}\right)\right)}^{-1}J_{\beta }\left(\theta_{0}\right){\left(H_{\beta }\left(\theta_{0}\right)\right)}^{-1}\right)}^{-1}\left({\hat{\theta }}_{c}^{\beta }-\theta_{0}\right).$$

For β=0, we get the classical Wald-type test statistic considered in the composite likelihood methods (see for instance [7]).

In the following theorem, we present the asymptotic null distribution of the family of the Wald-type test statistics $W_{n,\beta }^{0}.$

**Theorem** **2.** The asymptotic distribution of the Wald-type test statistics given in (14) is a chi-square distribution with p degrees of freedom.

The proof of this Theorem 2 is given in Appendix A.1.

**Theorem** **3.** Let $\theta^{*}$ be the true value of the parameter **θ**, with $\theta^{*}\neq \theta_{0}.$ Then, it holds:

$$\sqrt{n}\left(l\left({\hat{\theta }}_{c}^{\beta }\right)-l\left(\theta^{*}\right)\right)\underset{n\to \infty }{\overset{L}{⟶}}N\left(0,\sigma_{W_{\beta }^{0}}^{2}\left(\theta^{*}\right)\right),$$

*being:*

$$l\left(\theta \right)={\left(\theta -\theta_{0}\right)}^{T}{\left({\left(H_{\beta }\left(\theta_{0}\right)\right)}^{-1}J_{\beta }\left(\theta_{0}\right){\left(H_{\beta }\left(\theta_{0}\right)\right)}^{-1}\right)}^{-1}\left(\theta -\theta_{0}\right)$$

*and:*

(15) $$\sigma_{W_{\beta }^{0}}^{2}\left(\theta^{*}\right)=4{\left(\theta^{*}-\theta_{0}\right)}^{T}{\left({\left(H_{\beta }\left(\theta_{0}\right)\right)}^{-1}J_{\beta }\left(\theta_{0}\right){\left(H_{\beta }\left(\theta_{0}\right)\right)}^{-1}\right)}^{-1}\left(\theta^{*}-\theta_{0}\right).$$

The proof of the Theorem is outlined in Appendix A.2.

**Remark** **2.** Based on the previous result, we can approximate the power, $\beta_{W_{n}^{0}},$ of the Wald-type test statistics in $\theta^{*}$ by:

$$\begin{array}{ccc} \beta_{W_{n,\beta }^{0}}\left(\theta^{*}\right) & = & \mathrm{Pr}\left(W_{n,\beta }^{0}>\chi_{p,\alpha }^{2}/\theta =\theta^{*}\right)\\ & = & \mathrm{Pr}\left(\left.l\left({\hat{\theta }}_{c}^{\beta }\right)-l\left(\theta^{*}\right)>\frac{\chi_{p,\alpha }^{2}}{n}-l\left(\theta^{*}\right)\right|\theta =\theta^{*}\right)\\ & = & \mathrm{Pr}\left(\left.\sqrt{n}\left(l\left({\hat{\theta }}_{c}^{\beta }\right)-l\left(\theta^{*}\right)\right)>\sqrt{n}\left(\frac{\chi_{p,\alpha }^{2}}{n}-l\left(\theta^{*}\right)\right)\right|\theta =\theta^{*}\right)\\ & = & \mathrm{Pr}\left(\left.\sqrt{n}\frac{\left(l\left({\hat{\theta }}_{c}^{\beta }\right)-l\left(\theta^{*}\right)\right)}{\sigma_{W_{n,\beta }^{0}}\left(\theta^{*}\right)}>\frac{\sqrt{n}}{\sigma_{W_{n,\beta }^{0}}\left(\theta^{*}\right)}\left(\frac{\chi_{p,\alpha }^{2}}{n}-l\left(\theta^{*}\right)\right)\right|\theta =\theta^{*}\right)\\ & = & 1-\Phi_{n}\left(\frac{\sqrt{n}}{\sigma_{W_{n,\beta }^{0}}\left(\theta^{*}\right)}\left(\frac{\chi_{p,\alpha }^{2}}{n}-l\left(\theta^{*}\right)\right)\right), \end{array}$$

*where $\Phi_{n}$ is a sequence of distribution functions tending uniformly to the standard normal distribution function Φ(x).*

*It is clear that:*

$$\lim_{n\to \infty }\beta_{W_{n,\beta }^{0}}\left(\theta^{*}\right)=1$$

*for all $\alpha \in \left(0,1\right).$ Therefore, the Wald-type test statistics are consistent in the sense of Fraser.*

In many practical hypothesis testing problems, the restricted parameter space $\Theta_{0}\subset \Theta$ is defined by a set of *r* restrictions of the form:

(16) $$g\left(\theta \right)=0_{r}$$

on Θ, where $g:R^{p}\to R^{r}$ is a vector-valued function such that the p×r matrix:

(17) $$G\left(\theta \right)=\frac{\partial g{\left(\theta \right)}^{T}}{\partial \theta }$$

exists and is continuous in θ and rank $\left(G\left(\theta \right)\right)=r$; where $0_{r}$ denotes the null vector of dimension *r*.

Now, we are going to consider composite null hypotheses, $\Theta_{0}\subset \Theta$, in the way considered in (16), and our interest is in testing:

(18) $$H_{0}:\theta \in \Theta_{0}\;\mathrm{against}\;H_{1}:\theta \notin \Theta_{0}$$

on the basis of a random simple of size n, $X_{1},\ldots ,X_{n}.$

**Definition** **1.** *The family of Wald-type test statistics for testing (18) is given by:*

(19) $$W_{n,\beta }=ng{\left({\hat{\theta }}_{c}^{\beta }\right)}^{T}{\left[G{\left({\hat{\theta }}_{c}^{\beta }\right)}^{T}{\left(H_{\beta }\left({\hat{\theta }}_{c}^{\beta }\right)\right)}^{-1}J_{\beta }\left({\hat{\theta }}_{c}^{\beta }\right){\left(H_{\beta }\left({\hat{\theta }}_{c}^{\beta }\right)\right)}^{-1}G\left({\hat{\theta }}_{c}^{\beta }\right)\right]}^{-1}g\left({\hat{\theta }}_{c}^{\beta }\right),$$

*where the matrices $G\left(\theta \right),H_{\beta }\left(\theta \right)$ and $J_{\beta }\left(\theta \right)$ were defined in (17), (10) and (11), respectively, and the function g in (16).*

If we consider β=0, then ${\hat{\theta }}_{c}^{\beta }$ coincides with the CMLE, ${\hat{\theta }}_{c},$ of θ and ${\left(H_{\beta }\left({\hat{\theta }}_{c}\right)\right)}^{-1}J_{\beta }\left({\hat{\theta }}_{c}\right){\left(H_{\beta }\left({\hat{\theta }}_{c}\right)\right)}^{-1}$ with the inverse of the Fisher information matrix, and then, we get the classical Wald test statistic considered in the composite likelihood methods.

In the next theorem, we present the asymptotic distribution of $W_{n,\beta }.$

**Theorem** **4.** The asymptotic distribution of the Wald-type test statistics, given in (19), is a chi-square distribution with r degrees of freedom.

The proof of this Theorem is presented in Appendix A.3.

Consider the null hypothesis $H_{0}:\theta \in \Theta_{0}\subset \Theta$. By Theorem 4, the null hypothesis should be rejected if $\;W_{n,\beta }\geq \chi_{r,\alpha }^{2}$. The following theorem can be used to approximate the power function. Assume that $\theta^{*}\notin \Theta_{0}$ is the true value of the parameter, so that ${\hat{\theta }}_{c}^{\beta }\overset{a.s.}{\underset{n\to \infty }{⟶}}\theta^{*}$.

**Theorem** **5.** Let $\theta^{*}$ be the true value of the parameter, with $\theta^{*}\neq \theta_{0}.$ Then, it holds:

$$\sqrt{n}\left(l^{*}\left({\hat{\theta }}_{c}^{\beta }\right)-l^{*}\left(\theta^{*}\right)\right)\underset{n\to \infty }{\overset{L}{⟶}}N\left(0,\sigma_{W_{\beta }}^{2}\left(\theta^{*}\right)\right)$$

*being:*

$$l^{*}\left(\theta \right)=ng{\left(\theta \right)}^{T}{\left[G{\left(\theta_{0}\right)}^{T}{\left(H_{\beta }\left(\theta_{0}\right)\right)}^{-1}J_{\beta }\left(\theta_{0}\right){\left(H_{\beta }\left(\theta_{0}\right)\right)}^{-1}G\left(\theta_{0}\right)\right]}^{-1}g\left(\theta \right)$$

*and:*

(20) $$\sigma_{W_{\beta }}^{2}\left(\theta^{*}\right)={\left(\frac{\partial l^{*}\left(\theta \right)}{\partial \theta }\right)}_{\theta =\theta^{*}}^{T}{\left(H_{\beta }\left(\theta_{0}\right)\right)}^{-1}J_{\beta }\left(\theta_{0}\right){\left(H_{\beta }\left(\theta_{0}\right)\right)}^{-1}{\left(\frac{\partial l^{*}\left(\theta \right)}{\partial \theta }\right)}_{\theta =\theta^{*}}.$$

## 3. Numerical Example

In this section, we shall consider an example, studied previously by [8], in order to study the robustness of CMLE. The aim of this section is to clarify the different issues that were discussed in the previous sections.

Consider the random vector $Y={\left(Y_{1},Y_{2},Y_{3},Y_{4}\right)}^{T}$, which follows a four-dimensional normal distribution with mean vector $\mu ={\left(\mu_{1},\mu_{2},\mu_{3},\mu_{4}\right)}^{T}$ and variance-covariance matrix:

(21) $$\Sigma =\left(\begin{array}{cccc} 1 & \rho & 2\rho & 2\rho \\ \rho & 1 & 2\rho & 2\rho \\ 2\rho & 2\rho & 1 & \rho \\ 2\rho & 2\rho & \rho & 1 \end{array}\right),$$

i.e., we suppose that the correlation between $Y_{1}$ and $Y_{2}$ is the same as the correlation between $Y_{3}$ and $Y_{4}$. Taking into account that Σ should be semi-positive definite, the following condition is imposed: $-\frac{1}{5}\leq \rho \leq \frac{1}{3}$. In order to avoid several problems regarding the consistency of the CMLE of the parameter ρ (cf. [8]), we shall consider the composite likelihood function:

$$\mathrm{CL}\left(\theta ,y\right)=f_{A_{1}}\left(\theta ,y\right)f_{A_{2}}\left(\theta ,y\right),$$

where:

$$\begin{array}{cc} f_{A_{1}}\left(\theta ,y\right) & =f_{12}\left(\mu_{1},\mu_{2},\rho ,y_{1},y_{2}\right),\\ f_{A_{2}}\left(\theta ,y\right) & =f_{34}\left(\mu_{3},\mu_{4},\rho ,y_{3},y_{4}\right), \end{array}$$

where $f_{12}$ and $f_{34}$ are the densities of the marginals of Y, i.e., bivariate normal distributions with mean vectors ${\left(\mu_{1},\mu_{2}\right)}^{T}$ and ${\left(\mu_{3},\mu_{4}\right)}^{T}$, respectively, and common variance-covariance matrix:

$$\left(\begin{array}{cc} 1 & \rho \\ \rho & 1 \end{array}\right),$$

with densities given by:

$$f_{h,h+1}\left(\mu_{h},\mu_{h+1},\rho ,y_{h},y_{h+1}\right)=\frac{1}{2\pi \sqrt{1-\rho^{2}}}\exp\left\{-\frac{1}{2(1-\rho^{2})}Q\left(y_{h},y_{h+1}\right)\right\},\;h\in \left\{1,3\right\},$$

being:

$$Q\left(y_{h},y_{h+1}\right)={\left(y_{h}-\mu_{h}\right)}^{2}-2\rho \left(y_{h}-\mu_{h}\right)\left(y_{h+1}-\mu_{h+1}\right)+{\left(y_{h+1}-\mu_{h+1}\right)}^{2},\;h\in \left\{1,3\right\}.$$

By θ, we are denoting the parameter vector of our model, i.e, $\theta ={\left(\mu_{1},\mu_{2},\mu_{3},\mu_{4},\rho \right)}^{T}$. The system of equations that it is necessary to solve in order to obtain the CMDPDE:

$${\hat{\theta }}_{c}^{\beta }={\left({\hat{\mu }}_{1,c}^{\beta },{\hat{\mu }}_{2,c}^{\beta },{\hat{\mu }}_{3,c}^{\beta },{\hat{\mu }}_{4,c}^{\beta },{\hat{\rho }}_{c}^{\beta }\right)}^{T},$$

is given (see Appendix A.4) by:

(22) $$\frac{1}{n}\sum_{i=1}^{n}f_{12}{\left(\mu_{1},\mu_{2},\rho ,y_{1i},y_{2i}\right)}^{\beta -1}f_{34}{\left(\mu_{3},\mu_{4},\rho ,y_{3i},y_{4i}\right)}^{\beta }\left\{-\frac{1}{2\left(1-\rho^{2}\right)}\left[-2\left(y_{1i}-\mu_{1}\right)+2\rho \left(y_{2i}-\mu_{2}\right)\right]\right\}=0,$$

(23) $$\frac{1}{n}\sum_{i=1}^{n}f_{12}{\left(\mu_{1},\mu_{2},\rho ,y_{1i},y_{2i}\right)}^{\beta -1}f_{34}{\left(\mu_{3},\mu_{4},\rho ,y_{3i},y_{4i}\right)}^{\beta }\left\{-\frac{1}{2\left(1-\rho^{2}\right)}\left[-2\left(y_{2i}-\mu_{2}\right)+2\rho \left(y_{1i}-\mu_{1}\right)\right]\right\}=0,$$

(24) $$\frac{1}{n}\sum_{i=1}^{n}f_{12}{\left(\mu_{1},\mu_{2},\rho ,y_{1i},y_{2i}\right)}^{\beta -1}f_{34}{\left(\mu_{3},\mu_{4},\rho ,y_{3i},y_{4i}\right)}^{\beta }\left\{-\frac{1}{2\left(1-\rho^{2}\right)}\left[-2\left(y_{3i}-\mu_{3}\right)+2\rho \left(y_{4i}-\mu 4\right)\right]\right\}=0,$$

(25) $$\frac{1}{n}\sum_{i=1}^{n}f_{12}{\left(\mu_{1},\mu_{2},\rho ,y_{1i},y_{2i}\right)}^{\beta }f_{34}{\left(\mu_{3},\mu_{4},\rho ,y_{3i},y_{4i}\right)}^{\beta }\left\{-\frac{1}{2\left(1-\rho^{2}\right)}\left[-2\left(y_{4i}-\mu_{4}\right)+2\rho \left(y_{3i}-\mu_{3}\right)\right]\right\}=0$$

and:

(26) $$\frac{1}{n\beta }\sum_{i=1}^{n}\frac{\partial \mathrm{CL}{\left(\theta ,y_{i}\right)}^{\beta }}{\partial \rho }-\frac{\beta {\left(2\pi \right)}^{-2\beta }}{{\left(\beta +1\right)}^{3}}\frac{2\rho }{{\left(1-\rho^{2}\right)}^{\beta +1}}=0,$$

being:

$$\begin{array}{ccc} \frac{\partial \mathrm{CL}{\left(\theta ,y_{i}\right)}^{\beta }}{\partial \rho } & = & \frac{\rho }{1-\rho^{2}}\beta f_{12}{\left(\mu_{1},\mu_{2},\rho ,y_{1i},y_{2i}\right)}^{\beta }f_{34}{\left(\mu_{3},\mu_{4},\rho ,y_{3i},y_{4i}\right)}^{\beta }\\ & & \left\{2+\frac{1}{\rho }\left\{\left(y_{1i}-\mu_{1}\right)\left(y_{2i}-\mu_{2}\right)+\left(y_{3i}-\mu_{3}\right)\left(y_{4i}-\mu_{4}\right)\right\}\right.\\ & & \left.-\frac{1}{1-\rho^{2}}\left({\left(y_{1i}-\mu_{1}\right)}^{2}-2\rho \left(y_{1i}-\mu_{1}\right)\left(y_{2i}-\mu_{2}\right)+{\left(y_{2i}-\mu_{2}\right)}^{2}\right)\right.\\ & & \left.-\frac{1}{1-\rho^{2}}\left({\left(y_{3i}-\mu_{3}\right)}^{2}-2\rho \left(y_{3i}-\mu_{3}\right)\left(y_{4i}-\mu_{4}\right)+{\left(y_{4i}-\mu_{4}\right)}^{2}\right)\right\}. \end{array}$$

After some heavy algebraic manipulations specified in Appendix A.5, the sensitivity and variability matrices are given by:

(27) $$H_{\beta }\left(\theta \right)=\frac{C_{\beta }}{\left(\beta +1\right)\left(1-\rho^{2}\right)}\left(\begin{array}{ccccc} 1 & -\rho & 0 & 0 & 0\\ -\rho & 1 & 0 & 0 & 0\\ 0 & 0 & 1 & -\rho & 0\\ 0 & 0 & -\rho & 1 & 0\\ 0 & 0 & 0 & 0 & 2\frac{\left(\rho^{2}+1\right)+2\rho^{2}\beta^{2}}{\left(1-\rho^{2}\right)\left(1+\beta \right)} \end{array}\right)$$

and:

(28) $$J_{\beta }\left(\theta \right)=H_{2\beta }\left(\theta \right)-\xi_{\beta }\left(\theta \right)\xi_{\beta }{\left(\theta \right)}^{T},$$

where $C_{\beta }=\frac{1}{{\left(\beta +1\right)}^{2}}{\left(\frac{1}{{\left(2\pi \right)}^{2}\left(1-\rho^{2}\right)}\right)}^{\beta }$ and $\xi_{\beta }\left(\theta \right)={\left(0,0,0,0,\frac{2\rho \beta C_{\beta }}{\left(\beta +1\right)\left(1-\rho^{2}\right)}\right)}^{T}$.

### Simulation Study

A simulation study, developed by using the R statistical programming environment, is presented in order to study the behavior of the CMDPDE, as well as the behavior of the Wald-type test statistics based on them. The theoretical model studied in the previous example is considered. The parameters in the model are:

$$\theta ={\left(\mu_{1},\mu_{2},\mu_{3},\mu_{4},\rho \right)}^{T}$$

and we are interested in studying the behavior of the CMDPDE:

$${\hat{\theta }}_{c}^{\beta }={\left({\hat{\mu }}_{1,c}^{\beta },{\hat{\mu }}_{2,c}^{\beta },{\hat{\mu }}_{3,c}^{\beta },{\hat{\mu }}_{4,c}^{\beta },{\hat{\rho }}_{c}^{\beta }\right)}^{T}$$

as well as the behavior of the Wald-type test statistics for testing:

(29) $$H_{0}:\rho =\rho_{0}\;\mathrm{against}\;H_{1}:\rho \neq \rho_{0}.$$

Through *R* = 10,000 replications of the simulation experiment, we compare, for different values of β, the corresponding CMDPDE through the root of the mean square errors (RMSE), when the true value of the parameters is $\theta ={\left(0,0,0,0,\rho \right)}^{T}$ and ρ∈{−0.1,0,0.15}. We pay special attention to the problem of the existence of some outliers in the sample, generating 5% of the samples with $\tilde{\theta }={\left(1,3,-2,-1,\tilde{\rho }\right)}^{T}$ and $\tilde{\rho }\in \left\{-0.15,0.1,0.2\right\}$, respectively. Notice that, although the case ρ=0 has been considered; this case is less important taking into account the method of the theoretical model under consideration, and having the case of independent observations, the composite likelihood theory is useless. Results are presented in Table 1 and Table 2. Two points deserve our attention. The first one is that, as expected, RMSEs for contaminated data are always greater than RMSEs for pure data and that the RMSEs decrease when the sample size *n* increases. The second is that, while in pure data, RMSEs are greater for big values of β, when working with contaminated data, the CMDPDE with medium-low values of β (β∈{0.1,0.2,0.3}) present the best behavior in terms of efficiency. These statements are also true for larger levels of contamination, noting that, when larger percentages are considered, larger values of β are also considerable in terms of efficiency (see Table 3, Table 4 and Table 5 for contamination equal to 10%, 15% and 20%, respectively). Considering the mean absolute error (MAE) for the evaluation of the accuracy, we obtain similar results (Table 6).

For a nominal size α=0.05, with the model under the null hypothesis given in (29), the estimated significance levels for different Wald-type test statistics are given by:

$${\hat{\alpha }}_{n}^{\left(\beta \right)}\left(\rho_{0}\right)=\hat{\mathrm{Pr}}\left(W_{n}^{\beta }>\chi_{1,0.05}^{2}|H_{0}\right)=\frac{\sum_{i=1}^{R}I\left(W_{n,i}^{\beta }\right)>\chi_{1,0.05}^{2}\left|\rho_{0}\right)}{R},$$

with I(S) being the indicator function (with a value of one if *S* is true and zero otherwise). Empirical levels with the same previous parameter values are presented in Table 7 (pure data) and Table 8 (5% of outliers). While medium-high values of β are not recommended at all, CMLE is generally the best choice when working with pure data. However, the lack of robustness of the CMLE test is impressive, as can be seen in Table 8. The effect of contamination in medium-low values of β is much lighter, while for medium-high values of β, it can return to being deceptively beneficial.

For finite sample sizes and nominal size α=0.05, the simulated powers are obtained under $H_{1}$ in (29), when ρ∈{−0.1,0,0.1}, $\tilde{\rho }=0.2$ and $\rho_{0}=0.15$ (Table 9 and Table 10). The (simulated) power for different composite Wald-type test statistics is obtained by:

$$\beta_{n}^{\left(\beta \right)}\left(\rho_{0},\rho \right)=\mathrm{Pr}\left(W_{n}^{\beta }>\chi_{1,0.05}^{2}|H_{1}\right)\;\;\mathrm{and}\;\;{\hat{\beta }}_{n}^{\left(\lambda \right)}\left(\rho_{0},\rho \right)=\frac{\sum_{i=1}^{R}I\left(W_{n,i}^{\beta }>\chi_{1,0.05}^{2}|\rho_{0},\rho \right)}{R}.$$

As expected, when we get closer to the null hypothesis and when decreasing the sample sizes, the power decreases. With pure data, the best behavior is obtained with low values of β, and with this level of contamination (5%), the best results are obtained for medium values of β.

## 4. Conclusions

The likelihood function is the basis of the maximum likelihood method in estimation theory, and it also plays a key role in the development of log-likelihood ratio tests. However, it is not so tractable in many cases, in practice. Maximum likelihood estimators are based on the likelihood function, and they can be easily obtained; however, there are cases where they do not exist or they cannot be obtained. In such a case, composite likelihood methods constitute an appealing methodology in the area of estimation and testing of hypotheses. On the other hand, the distance or divergence based on methods of estimation and testing have increasingly become fundamental tools in the field of mathematical statistics. The work in [13] is the first, to the best of our knowledge, to link the notion of composite likelihood with divergence based on methods for testing statistical hypotheses.

In this paper, MDPDE are introduced, and they are exploited to develop Wald-type test statistics for testing simple or composite null hypotheses, in a composite likelihood framework. The validity of the proposed procedures is investigated by means of simulations. The simulation results point out the robustness of the proposed information theoretic procedures in estimation and testing, in the composite likelihood context. There are several areas where the notions of divergence and composite likelihood are crucial, including spatial statistics and time series analysis. These are areas of interest, and they will be explored elsewhere.

## Figures and Tables

**Table 1:** RMSEs for pure data.

	n=100			n=200			n=300		
	ρ=−0.1	ρ=0	ρ=0.15	ρ=−0.1	ρ=0	ρ=0.15	ρ=−0.1	ρ=0	ρ=0.15
β=0	0.0958	0.0950	0.0948	0.0683	0.0668	0.0666	0.0553	0.0552	0.0551
β=0.1	0.0972	0.0961	0.0966	0.0693	0.0676	0.0677	0.0560	0.0559	0.0561
β=0.2	0.1009	0.0991	0.1007	0.0718	0.0697	0.0704	0.0581	0.0575	0.0585
β=0.3	0.1061	0.1034	0.1062	0.0754	0.0727	0.0742	0.0612	0.0599	0.0619
β=0.4	0.1123	0.1087	0.1127	0.0797	0.0762	0.0787	0.0649	0.0628	0.0659
β=0.5	0.1195	0.1147	0.1200	0.0845	0.0803	0.0837	0.0691	0.0661	0.0702
β=0.6	0.1274	0.1215	0.1280	0.0898	0.0848	0.0892	0.0737	0.0697	0.0748
β=0.7	0.1361	0.1291	0.1369	0.0955	0.0897	0.0952	0.0786	0.0736	0.0797
β=0.8	0.1456	0.1374	0.1467	0.1015	0.0905	0.1016	0.0839	0.0778	0.0849

**Table 2:** RMSEs for contaminated data (5%).

	n=100			n=200			n=300		
	ρ=−0.1	ρ=0	ρ=0.15	ρ=−0.1	ρ=0	ρ=0.15	ρ=−0.1	ρ=0	ρ=0.15
β=0	0.1371	0.1336	0.1287	0.1210	0.1167	0.1113	0.1144	0.1098	0.1047
β=0.1	0.1105	0.1104	0.1081	0.0875	0.0874	0.0843	0.0778	0.0786	0.0748
β=0.2	0.1061	0.1053	0.1047	0.0783	0.0777	0.0759	0.0660	0.0669	0.0643
β=0.3	0.1091	0.1072	0.1083	0.0783	0.0766	0.0761	0.0646	0.0645	0.0635
β=0.4	0.1147	0.1118	0.1146	0.0814	0.0788	0.0798	0.0668	0.0657	0.0665
β=0.5	0.1215	0.1176	0.1220	0.0858	0.0823	0.0848	0.0703	0.0683	0.0709
β=0.6	0.1292	0.1242	0.1302	0.0907	0.0864	0.0905	0.0744	0.0716	0.0758
β=0.7	0.1375	0.1315	0.1391	0.0961	0.0911	0.0966	0.0790	0.0753	0.0810
β=0.8	0.1465	0.1396	0.1486	0.1018	0.0962	0.1031	0.0838	0.0794	0.0863

**Table 3:** RMSEs for contaminated data (10%).

	n=100			n=200			n=300		
	ρ=−0.1	ρ=0	ρ=0.15	ρ=−0.1	ρ=0	ρ=0.15	ρ=−0.1	ρ=0	ρ=0.15
β=0	0.2107	0.2052	0.2000	0.2003	0.1944	0.1884	0.1968	0.1911	0.1844
β=0.1	0.1500	0.1472	0.1436	0.1324	0.1305	0.1264	0.1259	0.1250	0.1204
β=0.2	0.1238	0.1229	0.1192	0.0991	0.0987	0.0951	0.0881	0.0898	0.0858
β=0.3	0.1173	0.1170	0.1139	0.0882	0.0871	0.0846	0.0735	0.0754	0.0726
β=0.4	0.1189	0.1187	0.1170	0.0872	0.0849	0.0845	0.0705	0.0714	0.0706
β=0.5	0.1237	0.1234	0.1234	0.0901	0.0868	0.0884	0.0721	0.0718	0.0734
β=0.6	0.1301	0.1296	0.1311	0.0944	0.0903	0.0938	0.0753	0.0742	0.0779
β=0.7	0.1375	0.1367	0.1396	0.0995	0.0947	0.1000	0.0793	0.0776	0.0831
β=0.8	0.1467	0.1446	0.1488	0.1050	0.0996	0.1064	0.0837	0.0814	0.0884

**Table 4:** RMSEs for contaminated data (15%).

	n=100			n=200			n=300		
	ρ=−0.1	ρ=0	ρ=0.15	ρ=−0.1	ρ=0	ρ=0.15	ρ=−0.1	ρ=0	ρ=0.15
β=0	0.2912	0.2854	0.2788	0.2835	0.2770	0.2713	0.2814	0.2757	0.2687
β=0.1	0.2036	0.1994	0.1951	0.1909	0.1874	0.1828	0.1871	0.185	0.1785
β=0.2	0.1530	0.1497	0.1453	0.1325	0.1306	0.1252	0.1252	0.1256	0.1181
β=0.3	0.1329	0.1295	0.1257	0.1049	0.1031	0.0976	0.0932	0.0945	0.0872
β=0.4	0.1287	0.1249	0.1229	0.0957	0.0931	0.0893	0.0805	0.0815	0.0763
β=0.5	0.1312	0.1272	0.1272	0.0949	0.0915	0.0902	0.0774	0.0777	0.0755
β=0.6	0.1367	0.1323	0.1343	0.0977	0.0936	0.0947	0.0784	0.0781	0.0788
β=0.7	0.1436	0.1389	0.1425	0.1019	0.0974	0.1005	0.0811	0.0804	0.0836
β=0.8	0.1514	0.1465	0.1514	0.1070	0.1020	0.1069	0.0847	0.0837	0.0888

**Table 5:** RMSEs for contaminated data (20%).

	n=100			n=200			n=300		
	ρ=−0.1	ρ=0	ρ=0.15	ρ=−0.1	ρ=0	ρ=0.15	ρ=−0.1	ρ=0	ρ=0.15
β=0	0.3725	0.3680	0.3612	0.3684	0.3618	0.3554	0.3661	0.3610	0.3534
β=0.1	0.2691	0.2657	0.2591	0.2625	0.2566	0.2506	0.2577	0.2547	0.2473
β=0.2	0.1949	0.1921	0.1831	0.1819	0.1766	0.1683	0.1742	0.1723	0.1624
β=0.3	0.1562	0.1537	0.1441	0.1345	0.1299	0.1204	0.1235	0.1222	0.1109
β=0.4	0.1419	0.1391	0.1316	0.1126	0.1082	0.1003	0.0987	0.0971	0.0876
β=0.5	0.1397	0.1366	0.1323	0.1050	0.1005	0.0962	0.0890	0.0867	0.0812
β=0.6	0.1430	0.1395	0.1383	0.1042	0.0996	0.0990	0.0866	0.0837	0.0828
β=0.7	0.1488	0.1450	0.1463	0.1066	0.1018	0.1043	0.0877	0.0843	0.0873
β=0.8	0.1560	0.1518	0.1552	0.1106	0.1056	0.1105	0.0905	0.0866	0.0927

**Table 6:** MAEs for pure and contaminated data (5%, 10%, 15% and 20%), n=100.

	Pure data		5%		10%		15%		20%	
	ρ=−0.1	ρ=0.15	ρ=−0.1	ρ=0.15	ρ=−0.1	ρ=0.15	ρ=−0.1	ρ=0.15	ρ=−0.1	ρ=0.15
β=0	0.076	0.076	0.190	0.179	0.371	0.342	0.626	0.574	0.954	0.877
β=0.1	0.077	0.077	0.167	0.163	0.289	0.277	0.464	0.437	0.697	0.652
β=0.2	0.081	0.080	0.165	0.163	0.263	0.257	0.388	0.372	0.551	0.520
β=0.3	0.085	0.085	0.172	0.170	0.264	0.260	0.370	0.359	0.495	0.473
β=0.4	0.090	0.090	0.181	0.180	0.275	0.272	0.377	0.370	0.489	0.474
β=0.5	0.095	0.095	0.192	0.192	0.290	0.289	0.394	0.391	0.504	0.496
β=0.6	0.101	0.102	0.204	0.204	0.308	0.308	0.416	0.416	0.528	0.527
β=0.7	0.108	0.109	0.218	0.218	0.328	0.329	0.441	0.444	0.558	0.561
β=0.8	0.115	0.116	0.232	0.233	0.349	0.351	0.468	0.474	0.590	0.599

**Table 7:** Levels for pure data.

	n=100			n=200			n=300		
	$\rho_{0}=-0.1$	$\rho_{0}=0$	$\rho_{0}=0.15$	$\rho_{0}=-0.1$	$\rho_{0}=0$	$\rho_{0}=0.15$	$\rho_{0}=-0.1$	$\rho_{0}=0$	$\rho_{0}=0.15$
β=0	0.067	0.059	0.070	0.068	0.046	0.062	0.072	0.045	0.075
β=0.1	0.067	0.060	0.072	0.062	0.046	0.070	0.085	0.045	0.079
β=0.2	0.072	0.061	0.084	0.069	0.051	0.084	0.097	0.049	0.102
β=0.3	0.081	0.062	0.093	0.084	0.053	0.100	0.112	0.051	0.121
β=0.4	0.094	0.069	0.099	0.103	0.055	0.111	0.127	0.055	0.142
β=0.5	0.105	0.071	0.111	0.118	0.056	0.122	0.149	0.051	0.155
β=0.6	0.122	0.083	0.129	0.131	0.062	0.136	0.167	0.051	0.165
β=0.7	0.135	0.088	0.141	0.139	0.063	0.146	0.181	0.055	0.177
β=0.8	0.153	0.099	0.158	0.151	0.071	0.156	0.198	0.056	0.179

**Table 8:** Levels for contaminated data (5%).

	n=100			n=200			n=300		
	$\rho_{0}=-0.1$	$\rho_{0}=0$	$\rho_{0}=0.15$	$\rho_{0}=-0.1$	$\rho_{0}=0$	$\rho_{0}=0.15$	$\rho_{0}=-0.1$	$\rho_{0}=0$	$\rho_{0}=0.15$
β=0	0.357	0.223	0.081	0.638	0.429	0.155	0.788	0.623	0.24 0
β=0.1	0.121	0.113	0.056	0.207	0.191	0.077	0.287	0.284	0.100
β=0.2	0.065	0.074	0.048	0.066	0.099	0.049	0.086	0.129	0.059
β=0.3	0.057	0.067	0.071	0.057	0.066	0.059	0.065	0.077	0.073
β=0.4	0.075	0.066	0.087	0.067	0.058	0.081	0.079	0.060	0.095
β=0.5	0.090	0.062	0.107	0.080	0.061	0.110	0.105	0.051	0.128
β=0.6	0.096	0.063	0.126	0.095	0.063	0.131	0.117	0.049	0.151
β=0.7	0.109	0.073	0.137	0.101	0.061	0.141	0.127	0.047	0.159
β=0.8	0.125	0.083	0.147	0.109	0.061	0.149	0.141	0.049	0.171

**Table 9:** Powers for pure data, $\rho_{0}=0.15$.

	n=100			n=200			n=300		
	ρ=−0.1	ρ=0	ρ=0.15	ρ=−0.1	ρ=0	ρ=0.15	ρ=−0.1	ρ=0	ρ=0.15
β=0	0.945	0.603	0.141	1	0.871	0.180	1	0.962	0.265
β=0.1	0.954	0.588	0.157	1	0.863	0.207	1	0.96	0.299
β=0.2	0.952	0.557	0.158	1	0.825	0.213	1	0.944	0.315
β=0.3	0.941	0.510	0.153	0.999	0.783	0.213	1	0.913	0.313
β=0.4	0.925	0.465	0.154	0.999	0.734	0.210	1	0.885	0.301
β=0.5	0.904	0.424	0.159	0.996	0.677	0.202	1	0.845	0.289
β=0.6	0.873	0.395	0.153	0.990	0.618	0.197	0.999	0.789	0.277
β=0.7	0.830	0.361	0.153	0.985	0.555	0.183	0.999	0.733	0.261
β=0.8	0.789	0.322	0.161	0.974	0.499	0.179	0.997	0.678	0.246

**Table 10:** Powers for contaminated data (5%), $\rho_{0}=0.15$.

	n=100			n=200			n=300		
	ρ=−0.1	ρ=0	ρ=0.15	ρ=−0.1	ρ=0	ρ=0.15	ρ=−0.1	ρ=0	ρ=0.15
β=0	0.424	0.090	0.029	0.746	0.141	0.030	0.919	0.246	0.037
β=0.1	0.716	0.222	0.041	0.954	0.397	0.029	0.994	0.569	0.037
β=0.2	0.838	0.333	0.071	0.989	0.555	0.075	0.999	0.744	0.096
β=0.3	0.881	0.383	0.105	0.993	0.633	0.121	0.999	0.803	0.161
β=0.4	0.879	0.393	0.129	0.993	0.642	0.150	0.999	0.809	0.213
β=0.5	0.865	0.381	0.135	0.992	0.621	0.168	0.999	0.797	0.241
β=0.6	0.836	0.357	0.149	0.984	0.583	0.174	0.998	0.769	0.252
β=0.7	0.808	0.332	0.146	0.980	0.531	0.173	0.997	0.713	0.256
β=0.8	0.773	0.309	0.152	0.961	0.487	0.173	0.995	0.657	0.243

## Abbreviations

The following abbreviations are used in this manuscript:

MLE	Maximum likelihood estimator
CMLE	Composite maximum likelihood estimator
DPD	Density power divergence
MDPDE	Minimum density power divergence estimator
CMDPDE	Composite minimum density power divergence estimator
RMSE	Root of mean square error
MAE	Mean absolute error

## Appendix A. Proof of the Results

### Appendix A.1. Proof of Theorem 2

The result follows in a straightforward manner because of the asymptotic normality of ${\hat{\theta }}_{c}^{\beta },$

$$\sqrt{n}\left({\hat{\theta }}_{c}^{\beta }-\theta_{0}\right)\underset{n\to \infty }{\overset{L}{⟶}}N\left(0,{\left(H_{\beta }\left(\theta_{0}\right)\right)}^{-1}J_{\beta }\left(\theta_{0}\right){\left(H_{\beta }\left(\theta_{0}\right)\right)}^{-1}\right).$$

### Appendix A.2. Proof of Theorem 3

A first order Taylor expansion of $l\left(\theta \right)$ at ${\hat{\theta }}_{c}^{\beta }$ around $\theta^{*}$ gives:

$$l\left({\hat{\theta }}_{c}^{\beta }\right)-l\left(\theta^{*}\right)={\left(\frac{\partial l\left(\theta \right)}{\partial \theta }\right)}_{\theta =\theta^{*}}\left({\hat{\theta }}_{c}^{\beta }-\theta^{*}\right)+o_{p}\left(∥{\hat{\theta }}_{c}^{\beta }-\theta^{*}∥\right).$$

Now, the result follows because the asymptotic distribution of $\left(l\left({\hat{\theta }}_{c}^{\beta }\right)-l\left(\theta^{*}\right)\right)$ coincides with the asymptotic distribution of $\sqrt{n}{\left(\frac{\partial l\left(\theta \right)}{\partial \theta }\right)}_{\theta =\theta^{*}}\left({\hat{\theta }}_{c}^{\beta }-\theta^{*}\right).$

### Appendix A.3. Proof of Theorem 4

We have:

$$\begin{array}{ccc} g({\hat{\theta }}_{c}^{\beta }) & = & g\left(\theta_{0}\right)+G{\left(\theta_{0}\right)}^{T}\left({\hat{\theta }}_{c}^{\beta }-\theta_{0}\right)+o_{p}\left(∥{\hat{\theta }}_{c}^{\beta }-\theta_{0}∥\right)\\ & = & G{\left(\theta_{0}\right)}^{T}\left({\hat{\theta }}_{c}^{\beta }-\theta_{0}\right)+o_{p}\left(∥{\hat{\theta }}_{c}^{\beta }-\theta_{0}∥\right), \end{array}$$

because $g\left(\theta_{0}\right)=0_{r}.$

Therefore:

$$\sqrt{n}g\left({\hat{\theta }}_{c}^{\beta }\right)\underset{n⟶\infty }{\overset{L}{⟶}}N\left(0,G_{\beta }{\left(\theta_{0}\right)}^{T}{\left(H_{\beta }\left(\theta_{0}\right)\right)}^{-1}J_{\beta }\left(\theta_{0}\right){\left(H_{\beta }\left(\theta_{0}\right)\right)}^{-1}G_{\beta }\left(\theta_{0}\right)\right)$$

because:

$$\sqrt{n}\left({\hat{\theta }}_{c}^{\beta }-\theta_{0}\right)\underset{n⟶\infty }{\overset{L}{⟶}}N\left(0,{\left(H_{\beta }\left(\theta_{0}\right)\right)}^{-1}J_{\beta }\left(\theta_{0}\right){\left(H_{\beta }\left(\theta_{0}\right)\right)}^{-1}\right).$$

Now:

$$W_{n,\beta }=ng{\left({\hat{\theta }}_{\beta }\right)}^{T}{\left[G{\left(\theta_{0}\right)}^{T}{\left(H_{\beta }\left(\theta_{0}\right)\right)}^{-1}J_{\beta }\left(\theta_{0}\right){\left(H_{\beta }\left(\theta_{0}\right)\right)}^{-1}G\left(\theta_{0}\right)\right]}^{-1}g\left({\hat{\theta }}_{\beta }\right)\underset{n⟶\infty }{\overset{L}{⟶}}\chi_{r}^{2}.$$

### Appendix A.4. CMDPE for the Numerical Example

The estimator ${\hat{\theta }}_{c}^{\beta }$ is obtained by maximizing Expression (6) with respect to θ. Firstly, we are going to get:

$$\begin{array}{ccc} \int_{R^{4}}\frac{\partial \mathrm{CL}{\left(\theta ,y\right)}^{1+\beta }}{\partial \theta }dy & = & \frac{\partial }{\partial \theta }\int_{R^{4}}\mathrm{CL}{\left(\theta ,y\right)}^{1+\beta }dy\\ & = & \frac{\partial }{\partial \theta }\int_{R^{4}}f_{12}{\left(\mu_{1},\mu_{2},\rho ,y_{1},y_{2}\right)}^{\beta +1}f_{34}{\left(\mu_{3},\mu_{4},\rho ,y_{3},y_{4}\right)}^{\beta +1}dy_{1}dy_{2}dy_{3}dy_{4}\\ & = & \frac{\partial }{\partial \theta }\left(\int_{R^{2}}f_{12}{\left(\mu_{1},\mu_{2},\rho ,y_{1},y_{2}\right)}^{\beta +1}dy_{1}dy_{2}\int_{R^{2}}f_{34}{\left(\mu_{3},\mu_{4},\rho ,y_{3},y_{4}\right)}^{\beta +1}dy_{3}dy_{4}\right). \end{array}$$

Based on [14] (p. 32):

$$\int_{R^{2}}f_{12}{\left(\mu_{1},\mu_{2},\rho ,y_{1},y_{2}\right)}^{\beta +1}dy_{1}dy_{2}=\int_{R^{2}}f_{34}{\left(\mu_{3},\mu_{4},\rho ,y_{3},y_{4}\right)}^{\beta +1}dy_{3}dy_{4}=\frac{{\left(1-\rho^{2}\right)}^{-\frac{\beta }{2}}}{\beta +1}{\left(2\pi \right)}^{-\beta }.$$

Then:

$$\int_{R^{4}}\frac{\partial \mathrm{CL}{\left(\theta ,y\right)}^{1+\beta }}{\partial \theta }dy=\frac{\partial }{\partial \theta }\int_{R^{4}}\mathrm{CL}{\left(\theta ,y\right)}^{1+\beta }dy=\frac{\partial }{\partial \theta }\frac{{\left(1-\rho^{2}\right)}^{-\beta }}{{\left(\beta +1\right)}^{2}}{\left(2\pi \right)}^{-2\beta }$$

and:

$$\frac{\partial }{\partial \mu_{i}}\frac{{\left(1-\rho^{2}\right)}^{-\beta }}{{\left(\beta +1\right)}^{2}}{\left(2\pi \right)}^{-2\beta }=0,\;i=1,2,3,4,$$

while:

$$\begin{array}{ccc} \frac{\partial }{\partial \rho }\frac{{\left(1-\rho^{2}\right)}^{-\beta }}{{\left(\beta +1\right)}^{2}}{\left(2\pi \right)}^{-2\beta } & = & \frac{\beta {\left(2\pi \right)}^{-2\beta }}{{\left(\beta +1\right)}^{2}}\frac{2\rho }{{\left(1-\rho^{2}\right)}^{\beta +1}}. \end{array}$$

Now, we are going to get:

$$\frac{1}{n\beta }\sum_{i=1}^{n}\frac{\partial \mathrm{CL}{\left(\theta ,y_{i}\right)}^{\beta }}{\partial \theta }$$

in order to obtain the CMDPDE, ${\hat{\theta }}_{c}^{\beta }$, by maximizing (6) with respect to θ.

We have,

$$\mathrm{CL}{\left(\theta ,y\right)}^{\beta }=f_{12}{\left(\mu_{1},\mu_{2},\rho ,y_{1},y_{2}\right)}^{\beta }f_{34}{\left(\mu_{3},\mu_{4},\rho ,y_{3},y_{4}\right)}^{\beta }.$$

Therefore,

$$\frac{\partial \mathrm{CL}{\left(\theta ,y_{i}\right)}^{\beta }}{\partial \mu_{1}}=\beta f_{12}{\left(\mu_{1},\mu_{2},\rho ,y_{1i},y_{2i}\right)}^{\beta -1}\left\{-\frac{1}{2\left(1-\rho^{2}\right)}\left[-2\left(y_{1i}-\mu_{1}\right)+2\rho \left(y_{2i}-\mu_{2}\right)\right]\right\}f_{34}{\left(\mu_{3},\mu_{4},\rho ,y_{3i},y_{4i}\right)}^{\beta }$$

and the expression:

$$\frac{1}{n\beta }\sum_{i=1}^{n}\frac{\partial \mathrm{CL}{\left(\theta ,y_{i}\right)}^{\beta }}{\partial \mu_{1}}=0$$

leads to the estimator of $\mu_{1}$, given by:

(A1) $$\frac{1}{n}\sum_{i=1}^{n}f_{12}{\left(\mu_{1},\mu_{2},\rho ,y_{1i},y_{2i}\right)}^{\beta -1}f_{34}{\left(\mu_{3},\mu_{4},\rho ,y_{3i},y_{4i}\right)}^{\beta }\left\{-\frac{1}{2\left(1-\rho^{2}\right)}\left[-2\left(y_{1i}-\mu_{1}\right)+2\rho \left(y_{2i}-\mu_{2}\right)\right]\right\}=0.$$

In a similar way:

$$\frac{\partial \mathrm{CL}{\left(\theta ,y_{i}\right)}^{\beta }}{\partial \mu_{2}}=\beta f_{12}{\left(\mu_{1},\mu_{2},\rho ,y_{1i},y_{2i}\right)}^{\beta -1}\left\{-\frac{1}{2\left(1-\rho^{2}\right)}\left[-2\left(y_{2i}-\mu_{2}\right)+2\rho \left(y_{1i}-\mu_{1}\right)\right]\right\}f_{34}{\left(\mu_{3},\mu_{4},\rho ,y_{3i},y_{4i}\right)}^{\beta },$$

$$\frac{\partial \mathrm{CL}{\left(\theta ,y_{i}\right)}^{\beta }}{\partial \mu_{3}}=\beta f_{12}{\left(\mu_{1},\mu_{2},\rho ,y_{1i},y_{2i}\right)}^{\beta }\left\{-\frac{1}{2\left(1-\rho^{2}\right)}\left[-2\left(y_{3i}-\mu_{3}\right)+2\rho \left(y_{4i}-\mu_{4}\right)\right]\right\}f_{34}{\left(\mu_{3},\mu_{4},\rho ,y_{3i},y_{4i}\right)}^{\beta -1}$$

and:

$$\frac{\partial \mathrm{CL}{\left(\theta ,y_{i}\right)}^{\beta }}{\partial \mu_{4}}=\beta f_{12}{\left(\mu_{1},\mu_{2},\rho ,y_{1i},y_{2i}\right)}^{\beta }\left\{-\frac{1}{2\left(1-\rho^{2}\right)}\left[-2\left(y_{4i}-\mu_{4}\right)+2\rho \left(y_{3i}-\mu_{3}\right)\right]\right\}f_{34}{\left(\mu_{3},\mu_{4},\rho ,y_{3i},y_{4i}\right)}^{\beta -1}.$$

Therefore, the equations:

$$\frac{1}{n\beta }\sum_{i=1}^{n}\frac{\partial \mathrm{CL}{\left(\theta ,y_{i}\right)}^{\beta }}{\partial \mu_{2}}=0,\;\frac{1}{n\beta }\sum_{i=1}^{n}\frac{\partial \mathrm{CL}{\left(\theta ,y_{i}\right)}^{\beta }}{\partial \mu_{3}}=0\;\;\mathrm{and}\;\;\frac{1}{n\beta }\sum_{i=1}^{n}\frac{\partial \mathrm{CL}{\left(\theta ,y_{i}\right)}^{\beta }}{\partial \mu_{4}}=0$$

lead to the estimators of $\mu_{2},\mu_{3}$ and $\mu_{4}$, which should be read as follows:

(A2) $$\frac{1}{n}\sum_{i=1}^{n}f_{12}{\left(\mu_{1},\mu_{2},\rho ,y_{1i},y_{2i}\right)}^{\beta -1}f_{34}{\left(\mu_{3},\mu_{4},\rho ,y_{3i},y_{4i}\right)}^{\beta }\left\{-\frac{1}{2\left(1-\rho^{2}\right)}\left[-2\left(y_{2i}-\mu_{2}\right)+2\rho \left(y_{1i}-\mu_{1}\right)\right]\right\}=0,$$

(A3) $$\frac{1}{n}\sum_{i=1}^{n}f_{12}{\left(\mu_{1},\mu_{2},\rho ,y_{1i},y_{2i}\right)}^{\beta -1}f_{34}{\left(\mu_{3},\mu_{4},\rho ,y_{3i},y_{4i}\right)}^{\beta }\left\{-\frac{1}{2\left(1-\rho^{2}\right)}\left[-2\left(y_{3i}-\mu_{3}\right)+2\rho \left(y_{4i}-\mu 4\right)\right]\right\}=0$$

and:

(A4) $$\frac{1}{n}\sum_{i=1}^{n}f_{12}{\left(\mu_{1},\mu_{2},\rho ,y_{1i},y_{2i}\right)}^{\beta }f_{34}{\left(\mu_{3},\mu_{4},\rho ,y_{3i},y_{4i}\right)}^{\beta }\left\{-\frac{1}{2\left(1-\rho^{2}\right)}\left[-2\left(y_{4i}-\mu_{4}\right)+2\rho \left(y_{3i}-\mu_{3}\right)\right]\right\}=0.$$

Now, it is necessary to get:

$$\begin{array}{ccc} \frac{\partial \mathrm{CL}{\left(\theta ,y_{i}\right)}^{\beta }}{\partial \rho } & = & \frac{\partial f_{12}{\left(\mu_{1},\mu_{2},\rho ,y_{1i},y_{2i}\right)}^{\beta }f_{34}{\left(\mu_{3},\mu_{4},\rho ,y_{3i},y_{4i}\right)}^{\beta }}{\partial \rho }\\ & = & \beta f_{12}{\left(\mu_{1},\mu_{2},\rho ,y_{1i},y_{2i}\right)}^{\beta -1}f_{34}{\left(\mu_{3},\mu_{4},\rho ,y_{3i},y_{4i}\right)}^{\beta }\frac{\partial f_{12}\left(\mu_{1},\mu_{2},\rho ,y_{1i},y_{2i}\right)}{\partial \rho }\\ & & +\beta f_{12}{\left(\mu_{1},\mu_{2},\rho ,y_{1i},y_{2i}\right)}^{\beta }f_{34}{\left(\mu_{3},\mu_{4},\rho ,y_{3i},y_{4i}\right)}^{\beta -1}\frac{\partial f_{34}\left(\mu_{3},\mu_{4},\rho ,y_{3i},y_{4i}\right)}{\partial \rho }. \end{array}$$

However, $\frac{\partial f_{12}\left(\mu_{1},\mu_{2},\rho ,y_{1i},y_{2i}\right)}{\partial \rho }$ is given by:

$$\begin{array}{cc} & \frac{1}{2\pi }\frac{\left(-1\right)}{\left(1-\rho^{2}\right)}\frac{\left(-2\rho \right)}{2{\left(1-\rho^{2}\right)}^{\frac{1}{2}}}\exp\left\{\frac{\left(-1\right)}{2\left(1-\rho^{2}\right)}\left[{\left(y_{1i}-\mu_{1}\right)}^{2}-2\rho \left(y_{1i}-\mu_{1}\right)\left(y_{2i}-\mu_{2}\right)+{\left(y_{2i}-\mu_{2}\right)}^{2}\right]\right\}\\ & +\frac{1}{2\pi {\left(1-\rho^{2}\right)}^{\frac{1}{2}}}\exp\left\{\frac{\left(-1\right)}{2\left(1-\rho^{2}\right)}\left[{\left(y_{1i}-\mu_{1}\right)}^{2}-2\rho \left(y_{1i}-\mu_{1}\right)\left(y_{2i}-\mu_{2}\right)+{\left(y_{2i}-\mu_{2}\right)}^{2}\right]\right\}\\ & \left[\frac{-\rho }{{\left(1-\rho^{2}\right)}^{2}}\left({\left(y_{1i}-\mu_{1}\right)}^{2}-2\rho \left(y_{1i}-\mu_{1}\right)\left(y_{2i}-\mu_{2}\right)+{\left(y_{2i}-\mu_{2}\right)}^{2}\right)+\frac{1}{\left(1-\rho^{2}\right)}\left(y_{1i}-\mu_{1}\right)\left(y_{2i}-\mu_{2}\right)\right]\\ = & \frac{\rho }{1-\rho^{2}}f_{12}\left(\mu_{1},\mu_{2},\rho ,y_{1i},y_{2i}\right)+f_{12}\left(\mu_{1},\mu_{2},\rho ,y_{1i},y_{2i}\right)\\ & \left[\frac{-\rho }{{\left(1-\rho^{2}\right)}^{2}}\left({\left(y_{1i}-\mu_{1}\right)}^{2}-2\rho \left(y_{1i}-\mu_{1}\right)\left(y_{2i}-\mu_{2}\right)+{\left(y_{2i}-\mu_{2}\right)}^{2}\right)+\frac{1}{\left(1-\rho^{2}\right)}\left(y_{1i}-\mu_{1}\right)\left(y_{2i}-\mu_{2}\right)\right]\\ = & f_{12}\left(\mu_{1},\mu_{2},\rho ,y_{1i},y_{2i}\right)\frac{\rho }{1-\rho^{2}}\left[1-\frac{1}{1-\rho^{2}}\left({\left(y_{1i}-\mu_{1}\right)}^{2}-2\rho \left(y_{1i}-\mu_{1}\right)\left(y_{2i}-\mu_{2}\right)+{\left(y_{2i}-\mu_{2}\right)}^{2}\right)\right.\\ & \left.+\frac{1}{\rho }\left(y_{1i}-\mu_{1}\right)\left(y_{2i}-\mu_{2}\right)\right]. \end{array}$$

In a similar way, $\frac{\partial f_{34}\left(\mu_{3},\mu_{4},\rho ,y_{3i},y_{4i}\right)}{\partial \rho }$ is given by:

$$\begin{array}{c} f_{34}\left(\mu_{3},\mu_{4},\rho ,y_{3i},y_{4i}\right)\frac{\rho }{1-\rho^{2}}\left[1-\frac{1}{1-\rho^{2}}\left({\left(y_{3i}-\mu_{3}\right)}^{2}-2\rho \left(y_{3i}-\mu_{3}\right)\left(y_{4i}-\mu_{4}\right)+{\left(y_{4i}-\mu_{4}\right)}^{2}\right)\right.\\ \left.+\frac{1}{\rho }\left(y_{3i}-\mu_{3}\right)\left(y_{4i}-\mu_{4}\right)\right]. \end{array}$$

Therefore,

(A5) $$\begin{array}{ccc} \frac{\partial \mathrm{CL}{\left(\theta ,y_{i}\right)}^{\beta }}{\partial \rho } & = & \frac{\rho }{1-\rho^{2}}\beta f_{12}{\left(\mu_{1},\mu_{2},\rho ,y_{1i},y_{2i}\right)}^{\beta }f_{34}{\left(\mu_{3},\mu_{4},\rho ,y_{3i},y_{4i}\right)}^{\beta }\\ & & \left\{2+\frac{1}{\rho }\left\{\left(y_{1i}-\mu_{1}\right)\left(y_{2i}-\mu_{2}\right)+\left(y_{3i}-\mu_{3}\right)\left(y_{4i}-\mu_{4}\right)\right\}\right.\\ & & \left.-\frac{1}{1-\rho^{2}}\left({\left(y_{1i}-\mu_{1}\right)}^{2}-2\rho \left(y_{1i}-\mu_{1}\right)\left(y_{2i}-\mu_{2}\right)+{\left(y_{2i}-\mu_{2}\right)}^{2}\right)\right.\\ & & \left.-\frac{1}{1-\rho^{2}}\left({\left(y_{3i}-\mu_{3}\right)}^{2}-2\rho \left(y_{3i}-\mu_{3}\right)\left(y_{4i}-\mu_{4}\right)+{\left(y_{4i}-\mu_{4}\right)}^{2}\right)\right\}. \end{array}$$

Therefore, the equation in relation to ρ is given by:

$$\frac{1}{n\beta }\sum_{i=1}^{n}\frac{\partial \mathrm{CL}{\left(\theta ,y_{i}\right)}^{\beta }}{\partial \rho }-\frac{1}{\beta +1}\int_{R^{m}}\frac{\partial \mathrm{CL}{\left(\theta ,y_{i}\right)}^{\beta +1}}{\partial \rho }\mathrm{dy}=0$$

being:

(A6) $$\int_{R^{m}}\frac{\partial \mathrm{CL}{\left(\theta ,y_{i}\right)}^{\beta +1}}{\partial \theta }\mathrm{dy}=\frac{\beta {\left(2\pi \right)}^{-2\beta }}{{\left(\beta +1\right)}^{2}}\frac{2\rho }{{\left(1-\rho^{2}\right)}^{\beta +1}}$$

and:

$$\frac{\partial \mathrm{CL}{\left(\theta ,y_{i}\right)}^{\beta }}{\partial \rho }$$

was given in (A5).

Finally,

$${\hat{\theta }}_{c}^{\beta }={\left({\hat{\mu }}_{1,c}^{\beta },{\hat{\mu }}_{2,c}^{\beta },{\hat{\mu }}_{3,c}^{\beta },{\hat{\mu }}_{4,c}^{\beta },{\hat{\rho }}_{c}^{\beta }\right)}^{T}$$

will be obtained as the solution of the system of equations given by (A1)–(A6).

### Appendix A.5. Computation of Sensitivity and Variability Matrices in the Numerical Example

We want to compute:

$$\begin{array}{cc} H_{\beta }\left(\theta \right) & =\int_{R^{m}}\mathrm{CL}{\left(\theta ,y\right)}^{\beta +1}u{\left(\theta ,y\right)}^{T}u\left(\theta ,y\right)dy\\ J_{\beta }\left(\theta \right) & =\int_{R^{m}}\mathrm{CL}{\left(\theta ,y\right)}^{2\beta +1}u{\left(\theta ,y\right)}^{T}u\left(\theta ,y\right)dy\\ & -\int_{R^{m}}\mathrm{CL}{\left(\theta ,y\right)}^{\beta +1}u\left(\theta ,y\right)dy\int_{R^{m}}{\left(u(\theta ,y)\right)}^{T}\mathrm{CL}{\left(\theta ,y\right)}^{\beta +1}dy. \end{array}$$

First of all, we can see that:

$$\begin{array}{cc} \mathrm{CL}{\left(\theta ,y\right)}^{\beta +1} & ={\left(f_{A_{1}}\left(\theta ,y\right)f_{A_{2}}\left(\theta ,y\right)\right)}^{\beta +1}\\ & ={\left(\frac{1}{2\pi \sqrt{1-\rho^{2}}}\exp\left\{-\frac{1}{2(1-\rho^{2})}Q\left(y_{1},y_{2}\right)\right\}\cdot \frac{1}{2\pi \sqrt{1-\rho^{2}}}\exp\left\{-\frac{1}{2(1-\rho^{2})}Q\left(y_{3},y_{4}\right)\right\}\right)}^{\beta +1}\\ & ={\left(\frac{1}{{\left(2\pi \right)}^{2}\left(1-\rho^{2}\right)}\right)}^{\beta +1}\exp\left\{-\frac{\beta +1}{2(1-\rho^{2})}\left[Q\left(y_{1},y_{2}\right)+Q\left(y_{3},y_{4}\right)\right]\right\}\\ & =\frac{1}{{\left(\beta +1\right)}^{2}}{\left(\frac{1}{{\left(2\pi \right)}^{2}\left(1-\rho^{2}\right)}\right)}^{\beta }\frac{{\left(\beta +1\right)}^{2}}{{\left(2\pi \right)}^{2}\left(1-\rho^{2}\right)}\exp\left\{-\frac{\beta +1}{2(1-\rho^{2})}\left[Q\left(y_{1},y_{2}\right)+Q\left(y_{3},y_{4}\right)\right]\right\}\\ & =C_{\beta }\cdot {\mathrm{CL}}_{\beta }^{*}, \end{array}$$

where $C_{\beta }=\frac{1}{{\left(\beta +1\right)}^{2}}{\left(\frac{1}{{\left(2\pi \right)}^{2}\left(1-\rho^{2}\right)}\right)}^{\beta }$ and ${\mathrm{CL}}_{\beta }^{*}={\mathrm{CL}}_{\beta }{\left(\theta ,y\right)}^{*}∼N\left(\mu ,\Sigma^{*}\right)$, with $\Sigma^{*}=\frac{1}{\beta +1}\Sigma$.

While $u\left(\theta ,y\right)=\frac{\partial \log\mathrm{CL}(\theta ,y)}{\partial \theta }$, we will denote as $u{\left(\theta ,y\right)}^{*}$ to $u{\left(\theta ,y\right)}^{*}=\frac{\partial \log{\mathrm{CL}}_{\beta }^{*}}{\partial \theta }$. Then:

(A7) $$\begin{array}{cc} u(\theta ,y) & =\frac{\partial \log\mathrm{CL}(\theta ,y)}{\partial \theta }=\frac{1}{\beta +1}\frac{\partial \log\mathrm{CL}{\left(\theta ,y\right)}^{\beta +1}}{\partial \theta }=\frac{1}{\beta +1}\frac{\partial \log(C_{\beta }\cdot {\mathrm{CL}}_{\beta }^{*})}{\partial \theta }\\ & =\frac{1}{\beta +1}\left(\frac{\partial \log C_{\beta }}{\partial \theta }+\frac{\partial \log{\mathrm{CL}}_{\beta }^{*}}{\partial \theta }\right)=\frac{1}{\beta +1}\left(\frac{\partial \log C_{\beta }}{\partial \theta }+u{\left(\theta ,y\right)}^{*}\right). \end{array}$$

Further,

(A8) $$\begin{array}{cc} \int_{R^{m}}\mathrm{CL}{\left(\theta ,y\right)}^{\beta +1}u\left(\theta ,y\right)dy & =\int_{R^{m}}\mathrm{CL}{\left(\theta ,y\right)}^{\beta +1}\frac{\partial \log\mathrm{CL}(\theta ,y)}{\partial \theta }dy=\int_{R^{m}}\mathrm{CL}{\left(\theta ,y\right)}^{\beta }\frac{\partial \mathrm{CL}(\theta ,y)}{\partial \theta }dy\\ & =\int_{R^{m}}\frac{1}{\beta +1}\frac{\partial \mathrm{CL}{\left(\theta ,y\right)}^{\beta +1}}{\partial \theta }dy=\frac{1}{\beta +1}\frac{\partial }{\partial \theta }\int_{R^{m}}\mathrm{CL}{\left(\theta ,y\right)}^{\beta +1}dy\\ & =\frac{1}{\beta +1}\frac{\partial C_{\beta }}{\partial \theta }={\left(0,0,0,0,\frac{2\rho \beta C_{\beta }}{\left(\beta +1\right)\left(1-\rho^{2}\right)}\right)}^{T}=\xi_{\beta }\left(\theta \right). \end{array}$$

Now:

(A9) $$\begin{array}{cc} & \int_{R^{4}}{\mathrm{CL}}^{\beta +1}u{\left(\theta ,y\right)}^{T}u\left(\theta ,y\right)dy\\ & =\int_{R^{4}}\left(C_{\beta }\cdot {\mathrm{CL}}_{\beta }^{*}\right)\frac{1}{{\left(\beta +1\right)}^{2}}{\left(\frac{\partial \log C_{\beta }}{\partial \theta }+u{\left(\theta ,y\right)}^{*}\right)}^{T}\left(\frac{\partial \log C_{\beta }}{\partial \theta }+u{\left(\theta ,y\right)}^{*}\right)dy\\ & =\frac{C_{\beta }}{{\left(\beta +1\right)}^{2}}\int_{R^{4}}\left[{\left(\frac{\partial \log C_{\beta }}{\partial \theta }\right)}^{T}\left(\frac{\partial \log C_{\beta }}{\partial \theta }\right){\mathrm{CL}}_{\beta }^{*}\right.\\ & \left.\;+{\mathrm{CL}}_{\beta }^{*}{\left(u{\left(\theta ,y\right)}^{*}\right)}^{T}\frac{\partial \log C_{\beta }}{\partial \theta }+{\mathrm{CL}}_{\beta }^{*}{\left(\frac{\partial \log C_{\beta }}{\partial \theta }\right)}^{T}u{\left(\theta ,y\right)}^{*}+{\mathrm{CL}}_{\beta }^{*}{\left(u{\left(\theta ,y\right)}^{*}\right)}^{T}u{\left(\theta ,y\right)}^{*}\right]dy\\ & =\frac{C_{\beta }}{{\left(\beta +1\right)}^{2}}\left[{\left(\frac{\partial \log C_{\beta }}{\partial \theta }\right)}^{T}\left(\frac{\partial \log C_{\beta }}{\partial \theta }\right)\int_{R^{4}}{\mathrm{CL}}_{\beta }^{*}dy+{\left(\int_{R^{4}}{\mathrm{CL}}_{\beta }^{*}u{\left(\theta ,y\right)}^{*}dy\right)}^{T}\left(\frac{\partial \log C_{\beta }}{\partial \theta }\right)\right.\\ & \left.\;+{\left(\frac{\partial \log C_{\beta }}{\partial \theta }\right)}^{T}\int_{R^{4}}{\mathrm{CL}}_{\beta }^{*}u{\left(\theta ,y\right)}^{*}dy+\int_{R^{4}}{\mathrm{CL}}_{\beta }^{*}{\left(u{\left(\theta ,y\right)}^{*}\right)}^{T}u{\left(\theta ,y\right)}^{*}dy\right]\\ & =\frac{C_{\beta }}{{\left(\beta +1\right)}^{2}}\left[K^{T}K+{\left(\int_{R^{4}}{\mathrm{CL}}_{\beta }^{*}u{\left(\theta ,y\right)}^{*}dy\right)}^{T}K+K^{T}\int_{R^{4}}{\mathrm{CL}}_{\beta }^{*}u{\left(\theta ,y\right)}^{*}dy+\int_{R^{4}}{\mathrm{CL}}_{\beta }^{*}{\left(u{\left(\theta ,y\right)}^{*}\right)}^{T}u{\left(\theta ,y\right)}^{*}dy\right], \end{array}$$

where $K=\frac{\partial \log C_{\beta }}{\partial \theta }=\left(0,0,0,0,\frac{2\rho \cdot \beta }{1-\rho^{2}}\right)$. However:

$$\begin{array}{cc} \int_{R^{4}}{\mathrm{CL}}_{\beta }^{*}u{\left(\theta ,y\right)}^{*}dy & =\int_{R^{4}}\left(\frac{1}{C_{\beta }}\mathrm{CL}{\left(\theta ,y\right)}^{\beta +1}\right)\left[\left(\beta +1\right)u\left(\theta ,y\right)-\frac{\partial \log C_{\beta }}{\partial \theta }\right]dy\\ & =\frac{\beta +1}{C_{\beta }}\left[\int_{R^{4}}\mathrm{CL}{\left(\theta ,y\right)}^{\beta +1}u\left(\theta ,y\right)dy\right]-\frac{K}{C_{\beta }}\int_{R^{4}}\mathrm{CL}{\left(\theta ,y\right)}^{\beta +1}dy\\ & =\frac{1}{C_{\beta }}\frac{\partial C_{\beta }}{\partial \theta }-K=K-K=0, \end{array}$$

and thus, (A9) can be expressed as:

$$\begin{array}{c} \int_{R^{4}}\mathrm{CL}{\left(\theta ,y\right)}^{\beta +1}u{\left(\theta ,y\right)}^{T}u\left(\theta ,y\right)dy=\frac{C_{\beta }}{{\left(\beta +1\right)}^{2}}\left[K^{T}K+\int_{R^{4}}{\mathrm{CL}}_{\beta }^{*}{\left(u{\left(\theta ,y\right)}^{*}\right)}^{T}u{\left(\theta ,y\right)}^{*}dy\right]. \end{array}$$

On the other hand, it is not difficult to prove that:

$$\int_{R^{4}}{\mathrm{CL}}_{\beta }^{*}{\left(u{\left(\theta ,y\right)}^{*}\right)}^{T}u{\left(\theta ,y\right)}^{*}dy=C\cdot \int_{R^{4}}\mathrm{CL}\left(\theta ,y\right)u{\left(\theta ,y\right)}^{T}u\left(\theta ,y\right)dy=C\cdot H_{0}\left(\theta \right),$$

where C=diag(β+1,β+1,β+1,β+1,1) and ([13]):

(A10) $$H_{0}\left(\theta \right)=\left(\begin{array}{ccccc} \frac{1}{1-\rho^{2}} & \frac{-\rho }{1-\rho^{2}} & 0 & 0 & 0\\ \frac{-\rho }{1-\rho^{2}} & \frac{1}{1-\rho^{2}} & 0 & 0 & 0\\ 0 & 0 & \frac{1}{1-\rho^{2}} & \frac{-\rho }{1-\rho^{2}} & 0\\ 0 & 0 & \frac{-\rho }{1-\rho^{2}} & \frac{1}{1-\rho^{2}} & 0\\ 0 & 0 & 0 & 0 & \frac{2(\rho^{2}+1)}{{\left(1-\rho^{2}\right)}^{2}} \end{array}\right).$$

Therefore,

$$H_{\beta }\left(\theta \right)=\frac{C_{\beta }}{{\left(\beta +1\right)}^{2}}\left[C\cdot H_{0}\left(\theta \right)+K^{T}K\right],$$

that is:

(A11) $$H_{\beta }\left(\theta \right)=\frac{C_{\beta }}{\left(\beta +1\right)\left(1-\rho^{2}\right)}\left(\begin{array}{ccccc} 1 & -\rho & 0 & 0 & 0\\ -\rho & 1 & 0 & 0 & 0\\ 0 & 0 & 1 & -\rho & 0\\ 0 & 0 & -\rho & 1 & 0\\ 0 & 0 & 0 & 0 & 2\frac{\left(\rho^{2}+1\right)+2\rho^{2}\beta^{2}}{\left(1-\rho^{2}\right)\left(1+\beta \right)} \end{array}\right).$$

Note that, for β=0, (A11) reduces to (A10).

On the other hand, the expression of the variability matrix $J_{\beta }\left(\theta \right)$ can be obtained from Expressions (27) and (A8) as:

(A12) $$J_{\beta }\left(\theta \right)=H_{2\beta }\left(\theta \right)-\xi_{\beta }\left(\theta \right)\xi_{\beta }{\left(\theta \right)}^{T}.$$
